# Magic mirror on the wall, which is the best meta-analysis one of all?

**DOI:** 10.1186/s13054-023-04564-w

**Published:** 2023-07-11

**Authors:** Fabio Silvio Taccone

**Affiliations:** Department of Intensive Care, Hôpital Universitaire de Bruxelles (HUB), Université Libre de Bruxelles (ULB), Route de Lennik, 808, 1070 Brussels, Belgium

A recent systematic review and meta-analysis has suggested that inducing hypothermia may have a positive effect on neurological outcomes in cardiac arrest (CA) patients, particularly when aiming for a temperature range of 32–34 °C [1]. However, I have concerns regarding the disparities between these conclusions and those reached by other recently published meta-analyses that have examined the same body of evidence [2–4]; these studies concluded that there is insufficient evidence to support the use of hypothermia for neuroprotection in this context. Furthermore, these findings have influenced the development of current guidelines, which now recommend actively preventing fever in CA patients [5].

A systematic review involves a comprehensive and unbiased synthesis of existing evidence on a specific research question or topic. The studies included in the four aforementioned meta-analyses share significant overlap, in particular for large randomized clinical trials (RCTs), with the except of a few small cohorts of patients (Table 1). The work conducted by the International Liaison Committee on Resuscitation aimed to provide a quantitative summary and estimate of the treatment effect [2]. However, this approach did not account for the heterogeneity in patient characteristics across studies or variations in target temperatures in the intervention and control groups. To address these limitations and enable comparisons of multiple temperature management strategies, a network meta-analysis was performed [3]; this approach allows for ranking interventions based on their effectiveness and informing treatment decisions, when direct comparisons were limited. Nonetheless, this method is also limited by the assumptions of data consistency among studies, which are necessary for valid analysis. In this context, a Bayesian meta-analysis was conducted, employing statistical methods that incorporate prior knowledge or beliefs into the analysis [4]. This approach provides estimates of treatment effects along with credible intervals that reflect the uncertainty in the estimates. However, it is important to note that the Bayesian meta-analysis requires the specification of prior distributions, which can introduce subjectivity, and data interpretation may be less accessible to readers. Interestingly, all three approaches converged on the same conclusion: there is currently insufficient evidence to support the use of hypothermia at a target temperature of 32–34 °C compared to active control of fever in reducing the risk of poor outcomes following CA.

**Table 1:** Included studies in the different systematic reviews

	Granfeldt [2]	Fernando [3]	Aneman [4]	Arrich [1]
Bernard (1)	X	X	X	X
Dankiewicz (2)	X	X	X	X
HACA (3)	X	X	X	X
Hachimi-Idrissi (4)	X	X	X	X
Hachimi-Idrissi (5)	X			X
Hachimi-Idrissi (6)	X			X
Kwon (7)				X
Lascarrou (8)	X	X	X	X
Laurent (9)	X	X	X	X
Mori (10)				X
Nielsen (11)			X	X
Zhang (12)	X			X
Callaway (13)	X			
Le Man (14)		X		
Lopez de Sa (15)		X		
Lopez de Sa (16)		X		

References list of the Table is presented in Additional file 1

The systematic review of Arrich et al. [1] included more studies (*n* = 12) than previous ones [2–4]. However, one study (Additional file 1: Ref 5 in Table) included some patients who had been previously included into another published trial (Additional file 1: Ref 4 in Table). Moreover, another trial (Additional file 1: Ref 12 in Table) is not available as full-text. Importantly, some of the included studies (Additional file 1: Ref 1, 4, 5, 6, 12, 13) had participants assigned to different treatment groups using an unclear randomization procedure or in a non-random manner (i.e., the day of the week). While RCTs are considered the gold standard for evaluating treatment interventions, this inaccurate random allocation of participants may result in imbalances between treatment groups, leading to confounding factors that can affect the validity and generalizability of the study results. As such, the role of such studies in evaluating the effects of hypothermia in CA patients remains questionable and might explain the different conclusions of these meta-analyses. However, there are also some additional important considerations. Firstly, the presence of substantial uncertainty in the results suggests the possibility of a minimally clinically important difference, which may have been influenced by patient selection. Therefore, it cannot be excluded that a subset of patients may indeed benefit from hypothermia treatment. Secondly, there is a need for improved patient selection criteria to identify those individuals at high-risk of moderate brain injury, i.e., those who are not too mildly affected, as they may not require a specific neuroprotective strategy, and not too severely affected, as they may have poor prognosis regardless of any intervention. Thirdly, it is important to note that the majority of studies supporting the effectiveness of hypothermia were published over 15 years ago. Since then, there have been advancements in the overall care provided to CA patients, which may have potentially diminished the impact of such intervention in this particular context. A chronological cumulative meta-analysis would have better estimated how more recent and methodologically robust trials have influenced the overall evidence on this topic.

While it remains crucial to obtain a deeper understanding of the optimal strategies for temperature management to improve outcomes in patients after cardiac arrest, recent large trials have indicated that the results of hypothermia in unselected populations of CA patients are ineffective and the conclusions of this recent meta-analysis should not influence existing guidelines.

## Supplementary Information

**Additional file 1.** References for Table 1.

## Data Availability

Not applicable.
