# Supplementary material for: Magic mirror on the wall, which is the best meta-analysis one of all?
Source: Crit Care. 2023 Jul 11;27:280. doi: 10.1186/s13054-023-04564-w (PMC10334615; doi:10.1186/s13054-023-04564-w)
Supplement: Supplementary file 1 — Additional file 1. References for Table 1. [file 13054_2023_4564_MOESM1_ESM.docx]

**Additional file 1:** References for the Table 1.

1. Bernard SA, Gray TW, Buist MD, Jones BM, Silvester W, Gutteridge G, et al. Treatment of comatose survivors of out-of-hospital cardiac arrest with induced hypothermia. N Engl J Med 2002; 346(8):557-63.
2. Dankiewicz J, Cronberg T, Lilja G, Jakobsen JC, Levin H, Ullén S, et al. Hypothermia versus normothermia after out-of-hospital cardiac arrest. N Engl J Med 2021;384(24):2283-94.
3. Hypothermia after Cardiac Arrest Study Group. Mild therapeutic hypothermia to improve the neurologic outcome after cardiac arrest. N Engl J Med 2002; 346(8):549-56.
4. Hachimi-Idrissi S, Corne L, Ebinger G, Michotte Y, Huyghens L. Mild hypothermia induced by a helmet device: a clinical feasibility study. Resuscitation 2001;51(3):275-81.
5. Hachimi-Idrissi S, Zizi M, Nguyen DN, Schiettecate J, Ebinger G, Michotte Y, et al. The evolution of serum astroglial S-100 beta protein in patients with cardiac arrest treated with mild hypothermia. Resuscitation 2005;64(2):187-92.
6. Hachimi-Idrissi S, Zizi M, Nguyen DN, Schiettecate J, Ebinger G, Michotte Y, et al. The evolution of serum astroglial S-100 beta protein in patients with cardiac arrest treated with mild hypothermia. Resuscitation 2005;64(2):187-92.
7. Kwon WY, Jung YS, Suh GJ, Kim T, Kwak H, Kim T, et al. Regional cerebral oxygen saturation in cardiac arrest survivors undergoing targeted temperature management 36 °C versus 33°C: a randomized clinical trial. Resuscitation 2021;167:362-71.
8. Lascarrou JB, Merdji H, Le Gouge A, Colin G, Grillet G, Girardie P, et al. Targeted temperature management for cardiac arrest with non-shockable rhythm. N Engl J Med 2019; 381(24):2327-37.
9. Laurent I, Adrie C, Vinsonneau C, Cariou A, Chiche JD, Ohanessian A, et al. High-volume hemofiltration after out-of-hospital cardiac arrest: a randomized study. J Am Coll Cardiol 2005;46(3):432-7.
10. Mori K, Takeyama Y, Itoh Y, Nara S, Yoshida M, Ura H, et al. A multivariate analysis of prognostic factors in survivors of outof- hospital cardiac arrest with brain hypothermia. Crit Care Med 2000; 28:A168.
11. Nielsen N, Wetterslev J, Cronberg T, Erlinge D, Gasche Y, Hassager C, et al. Targeted temperature management at 33°C versus 36°C after cardiac arrest. N Engl J Med 2013; 369(23):2197-206.
12. Zhang J. Comparison and evaluation of brain-functional protection with mild hypothermia technique and normal temperature technique. Chin J Clin Rehabil 2005; 9(45):136-8.
13. Callaway CW, Tadler SC, Katz LM, Lipinski CL, Brader E. Feasibility of external cranial cooling during out-of-hospital cardiac arrest. Resuscitation 2002; 52(2):159–65.
14. Le May M, Osborne C, Russo J, So D, Chong AY, Dick A, et al. Effect of Moderate vs Mild Therapeutic Hypothermia on Mortality and Neurologic Outcomes in Comatose Survivors of Out-of-Hospital Cardiac Arrest: The CAPITAL CHILL Randomized Clinical Trial. JAMA. 2021; 326(15):1494-1503.
15. Lopez-de-Sa E, Rey JR, Armada E, Salinas P, Viana-Tejedor A, Espinosa-Garcia S, et al. Hypothermia in comatose survivors from out-of-hospital cardiac arrest: pilot trial comparing 2 levels of target temperature. Circulation 2012; 126:2826–2833.
16. Lopez-de-Sa E, Juarez M, Armada E, Sanchez-Salado JC, Sanchez PL, Loma-Osorio P, Sionis A, Monedero MC, Martinez-Sellés M, Martín-Benitez JC, Ariza A, Uribarri A, et al. A multicentre randomized pilot trial on the effectiveness of different levels of cooling in comatose survivors of out-of-hospital cardiac arrest: the FROST-I trial. Intensive Care Med 2018; 44:1807–1815.
